# Valproate Targets Mammalian Gastrulation Impairing Neural Tissue Differentiation and Development of the Placental Source In Vitro

**DOI:** 10.3390/ijms23168861

**Published:** 2022-08-09

**Authors:** Ana Katušić-Bojanac, Milvija Plazibat, Marta Himelreich-Perić, Katarina Eck-Raković, Jure Krasić, Nino Sinčić, Gordana Jurić-Lekić, Davor Ježek, Floriana Bulić-Jakuš

**Affiliations:** 1 Scientific Centre of Excellence for Reproductive and Regenerative Medicine, Unit for Biomedical Investigation of Reproduction and Development, School of Medicine, University of Zagreb, 10000 Zagreb, Croatia; 2 Department of Biology, School of Medicine, University of Zagreb, 10000 Zagreb, Croatia; 3 Pediatrics Department, General Hospital Zabok, 49210 Zabok, Croatia; 4 Dental Medicine and Health, School of Medicine, University of Osijek, 31000 Osijek, Croatia; 5 Anesthesiology and Intensive Care Department, Karolinska University Hospital, 14152 Stockholm, Sweden; 6 Department of Histology and Embryology, School of Medicine, University of Zagreb, 10000 Zagreb, Croatia

**Keywords:** valproate, HDACi, neural tissue, ectoplacental cone, embryo

## Abstract

The teratogenic activity of valproate (VPA), an antiepileptic and an inhibitor of histone deacetylase (HDACi), is dose-dependent in humans. Previous results showed that VPA impairs in vitro development and neural differentiation of the gastrulating embryo proper. We aimed to investigate the impact of a lower VPA dose in vitro and whether this effect is retained in transplants in vivo. Rat embryos proper (E9.5) and ectoplacental cones were separately cultivated at the air-liquid interface with or without 1 mM VPA. Embryos were additionally cultivated with HDACi Trichostatin A (TSA), while some cultures were syngeneically transplanted under the kidney capsule for 14 days. Embryos were subjected to routine histology, immunohistochemistry, Western blotting and pyrosequencing. The overall growth of VPA-treated embryos in vitro was significantly impaired. However, no differences in the apoptosis or proliferation index were found. Incidence of the neural tissue was lower in VPA-treated embryos than in controls. TSA also impaired growth and neural differentiation in vitro. VPA-treated embryos and their subsequent transplants expressed a marker of undifferentiated neural cells compared to controls where neural differentiation markers were expressed. VPA increased the acetylation of histones. Our results point to gastrulation as a sensitive period for neurodevelopmental impairment caused by VPA.

## 1. Introduction

In individuals exposed to valproate (VPA) during intrauterine development, fetal valproate syndrome (FVS), characterized by a pattern of major and minor malformations, can be diagnosed. Recently, the European Reference Network for Congenital Malformations and Intellectual Disability proposed the term “fetal valproate spectrum disorder” because of the variety of individual presentations where even those that lack major malformations and typical facial dysmorphogenesis may still have an increased risk of reduced IQ and autistic spectrum disorder (ASD) [1]. Higher doses of VPA were associated with a poorer cognitive outcome in children compared to other antiepileptics, where this outcome was not convincingly dose-dependent [2]. Recently, a ban on using VPA for migraine or bipolar disorder during pregnancy and for treating epilepsy during pregnancy unless there is no other effective treatment was agreed upon by EU Member State and some non-member states representatives. Where VPA has to be applied, a pregnancy prevention program has been recommended [3].

Antiepileptic properties of VPA depend on inhibition of Gamma Amino Butyrate (GABA) transaminobutyrate and ion channels. On the other hand, VPA is an epigenetic agent, a histone deacetylase inhibitor (HDACi) that acetylates histones and thus affects chromatin state and gene expression [4,5]. Indeed, some chromatinopathies, a heterogeneous group of Mendelian disorders, were found to be phenocopies of FVSD [6], implying the necessity for further research on epigenetic mechanisms such as posttranslational histone modifications during development. In a seven-day in vitro culture of the mouse gastrulating embryo proper, HDACi VPA downregulated and the other HDACi trichostatin A (TSA) upregulated the expression of pluripotency and the three germ-layer-differentiation genes [7]. Changes in gene activity associated with VPA can lead to changes in the cell cycle, differentiation, and apoptosis [8]. For example, VPA and TSA can activate tumor suppressor genes such as p21 and p53, p27, and p57 through inhibition of class I HDAC activity in a hepatocellular carcinoma cell line [9], and VPA positively affects also the intrinsic mitochondrial apoptotic pathway, leading treated cells to apoptosis [10].

In humans, VPA may cause IQ decline, autism spectrum disorders (ASD), attention-deficit hyperactivity disorder (ADHD) symptoms, spina bifida, cleft palate, cardiac abnormalities, and hypospadias, which is generally associated with the therapy during the first trimester of pregnancy [11]. Research in animal models is the only way to find the exact developmental time windows for the sensitivity to VPA. A recent systematic review of the research in rodent ASD models has pinpointed the gestational age of 12.5 ± 1 day, concomitant with the window of neural tube closure, as sensitive to the influence of VPA [12]. Among the parameters assessed in the rat model of ASD, the research of Kim et al. showed that the sociability index was similarly low after the treatment on day 9.5 of gestation, although it did not show a statistically significant difference from the treatment on day 12.5 of gestation [13], which points to the earlier stages of development that might be sensitive to VPA.

Indeed, the gastrulation that takes place at E7.5 in the mouse [14] and 9.5 days in the rat is known as the most sensitive period for the activity of teratogenic factors because at that stage the embryo has lost the plasticity that characterizes preimplantation [15,16,17] and postimplantation development before mesoderm formation [18]. At gastrulation, three germ layers (ectoderm, mesoderm, and endoderm) form to give rise to all tissues and organs in the body through a series of “inductive interactions” regulated by signaling pathways dependent on the proper differential gene activity. Several mammalian 3D in vitro models of “gastruloids” built from a variety of embryonic stem cells (ESC or iPSC) have recently been developed with the potential to support research on embryotoxic and teratogenic substances [19]. From our experience, a natural gastrulating embryo 3D model in vitro has shown valuable results in research of embryotoxic/teratogenic substances such as a DNA demethylating drug 5-azacytidine or retinoic acid [20,21].

Moreover, such in vitro models align with the attempts to minimize research on living animals by using in vitro alternatives as proposed in the Directive 2010/63/EU on protecting animals used for scientific purposes [22]. In vivo results on pregnant rodents, conducted to predict the teratogenic potential of chemical agents, depend on many factors such as the species-specificity pharmacokinetics, genetics, route of administration, age, diet, and environmental factors. On the other hand, in vitro approaches dealing with the critical developmental phases, devoid of the complicated and confounding maternal influences, may provide a clearer insight into the direct impact of a teratogen on the embryo [23]. Recently, it was shown that VPA alters the expression of placental carriers in a human placental cell line in vitro [24], in both mid-gestation and late gestation of the mouse [25], and in perfused human placenta [26], which all points to the placenta as a novel target of VPA for adverse fetal outcomes.

Our recent in vitro research on 9.5-days-old gastrulating rat embryos proper grown in vitro for 14 days showed that VPA acetylated histones enhanced apoptosis and impaired overall growth, proliferation, survival, and incidence of neural tissue. No teratomas developed after in vivo transplantation of pre-cultivated embryos [27]. Therefore, in this biological system, we showed both the embryotoxic and teratoma-antitumor activity of VPA. Indeed, HDACis have been used today for treating hematological malignancies or are in different phases of clinical trials [28] for solid tumors such as breast cancer [29].

Because it is known that the impact of VPA on major congenital malformations [30] and neurodevelopmental outcomes is dose-dependent [2], we now aimed to investigate the impact of VPA on the gastrulating rat embryo proper grown in vitro with a lower dose (1 mM) of VPA than we previously used (2 mM) [27]. Cultivation in vitro was combined with subsequent syngeneic transplantation in vivo to assess the extent of the remaining developmental potential. Additionally, we investigated in vitro/in vivo the development of the early placental source—the ectoplacental cone of the gastrulating rat embryo. Our results showed that the lower dose of VPA impaired overall growth and neural differentiation in vitro and that the effect on neural development is retained in transplants in vivo. At the same time, VPA also impaired the development of ectoplacental cones.

## 2. Results

### 2.1. Survival, Overall Growth, and Differentiation in Embryos Proper Cultivated with Valproate

When explanted gastrulating rat embryos proper were cultivated for 14 days in a serum-supplemented organ-culture model in vitro, all samples cultivated with valproate (VPA) (1 mM) survived in the same way as samples cultivated without VPA (Table 1).

**Figure 1:**
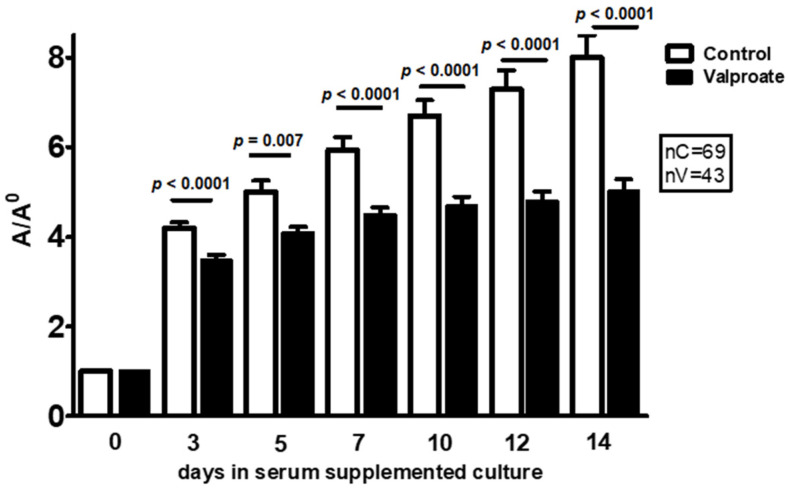
Influence of valproate (VPA) (1 mM) on the overall embryo proper growth during a 14 days culture period in the serum-supplemented medium. A/A0 = area on a day of culture/area on the day of plating, arithmetic mean ± SEM. Student *t*-test.

However, already from the third day of culture, the overall growth of embryos treated with VPA was significantly lower than in controls grown without VPA (Figure 1).

**Figure 2:**
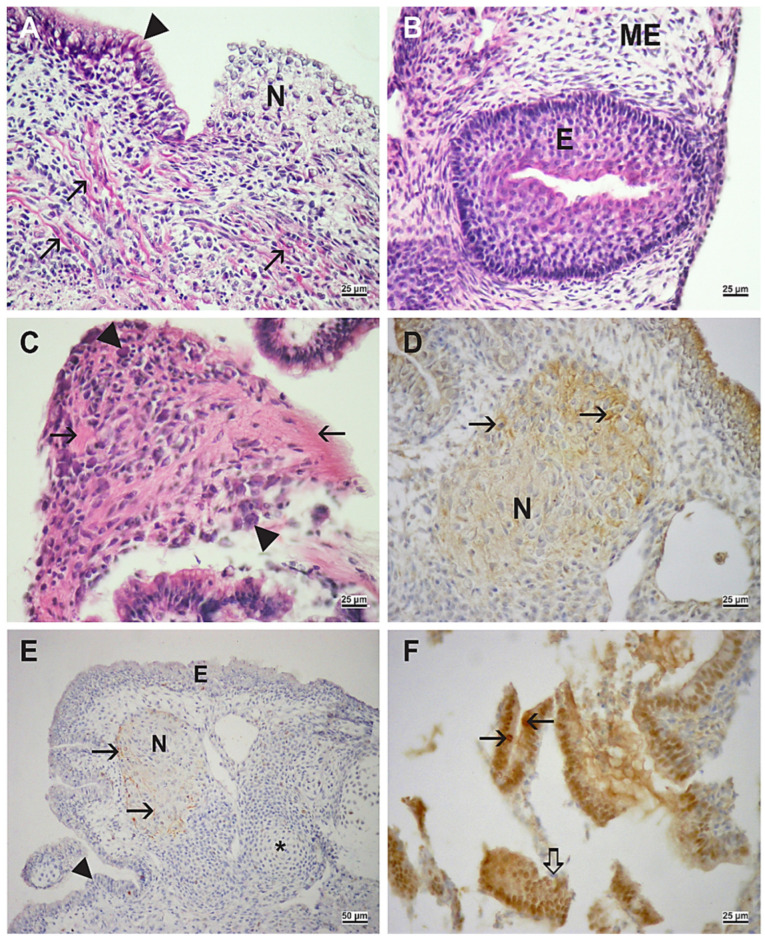
Embryo differentiation in the serum-supplemented medium (MEM with serum). (**A**) Ciliated pseudostratified columnar epithelium (arrowhead), neural tissue (N), muscle (M). (**B**) Epidermis (E), mesenchyme (ME). (**C**) Neuropil (arrow), ganglion cell (arrowhead). (**A**–**C**) Hematoxylin-eosin (HE) stain. (**D**) Neurofilament (NF)-positive signal (arrow), neural tissue (N). (**E**) Glial fibrillary acidic protein (GFAP)-positive signal (arrow), neural tissue (N), epidermis (E), cartilage (asterisk), columnar epithelium (arrowhead). (**F**) Hepatocyte nuclear factor 4 alpha (HNF4α)-positive cells (arrow), negative inner control (thick arrow). (**D**–**F**) Immunohistochemistry (IHC), DAB, contrasted by hematoxylin. Scale bar on insets (**A**–**D**,**F**) 25 µm, and on inset (**E**) 50 µm.

**Figure 3:**
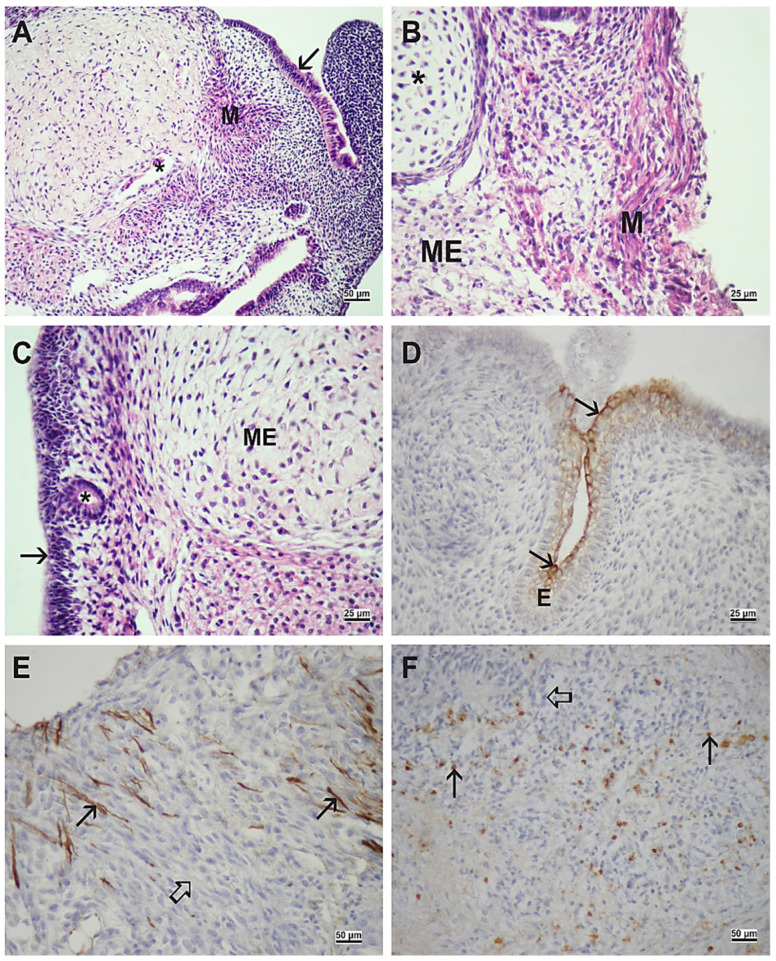
Embryo differentiation in the serum-supplemented medium with 1 mM VPA. (**A**) muscle (M), ciliated pseudostratified columnar epithelium (arrow), blood vessel (asterisk). (**B**) muscle (M), cartilage (asterisk), mesenchyme (ME). (**C**) pseudostratified columnar epithelium (arrow), mesenchyme (ME), neuroepithelium (asterisk). (**A**–**C**) HE stain. (**D**) cytokeratin-positive signal (arrow) in superficial layers of the epidermis. (**E**) nestin-positive signal (arrow), negative inner control (hollow arrow). (**F**) HNF4α-positive cells (arrow), negative inner control (hollow arrow). (**D**–**F**) IHC, DAB, contrasted by hematoxylin. Scale bar on insets (**A**,**E**,**F**) 25 µm, and on insets (**B**–**D**) 50 µm.

At the end of the culture period, cultivated embryos were reminiscent of teratoma-like structures containing various tissues, derivatives of the three germ layers found in the gastrulating embryo proper itself. In all embryos, ectodermal (epidermis, neural tissue), mesodermal (immature mesenchyme, cartilage, and muscle), and endodermal (columnar epithelium) derivatives developed (Figure 2 and Figure 3).

In control embryos, neural tissue could be recognized by the neuropil with fully differentiated neural cells expressing neurofilaments (NF) and cells of the glia expressing glial fibrillary acidic proteins (GFAP) (Figure 2). In contrast, embryos cultivated with VPA expressed only nestin, a marker of neural stem cells (Figure 3). Therefore, the degree of neural differentiation was lower in VPA-treated embryos. In both groups of embryos, the transcription factor hepatocyte nuclear factor 4 alpha (HNF4α), as a marker of definitive endoderm differentiation, was expressed in the columnar epithelium (Figure 2) or scattered throughout the tissues (Figure 3).

**Figure 4:**
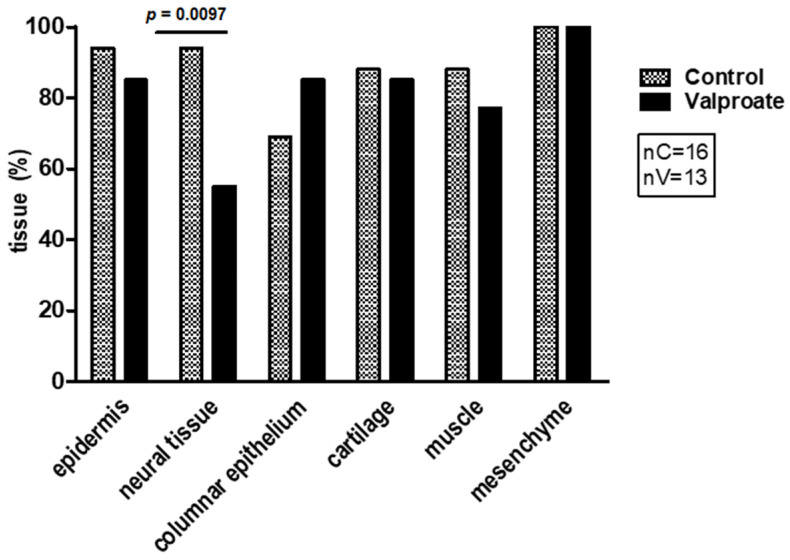
Influence of 1 mM VPA on the percentage of differentiated tissues in cultivated explants during a 14-day-long culture period in the serum-supplemented medium. Arithmetic mean ± SEM. Student *t*-test.

The neural tissue incidence assessment also showed that VPA significantly impaired neural tissue differentiation (Figure 4).

### 2.2. Overall Growth, and Differentiation in Embryos Proper Cultivated with Trichostatin A

**Figure 5:**
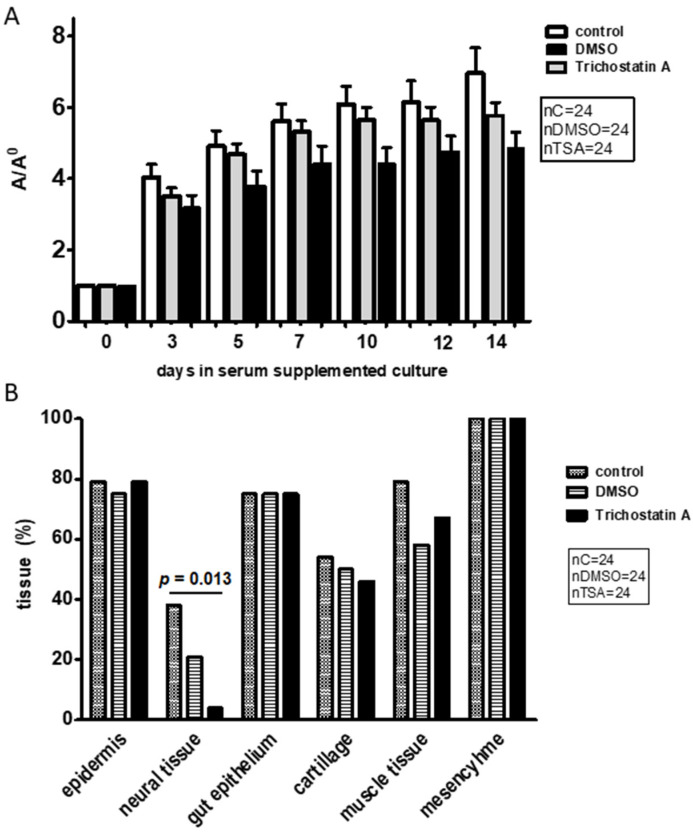
Growth and differentiation of embryos proper with trichostatin A (TSA). (**A**) Overall growth compared over 14 days in serum-supplemented cultures (control), DMSO, and TSA. 24 embryos/group. A/A0 = area on a day of culture/area on the day of plating, arithmetic mean ± SEM, ANOVA. (**B**) Frequency of differentiated tissues in serum-supplemented culture conditions with TSA and DMSO. χ^2^ = 6189.

Knowing that VPA is an HDACi, we also investigated the impact of another HDACi, trichostatin A (TSA), on survival, overall growth, and differentiation in cultivated embryos proper (Figure 5). As TSA had to be dissolved with dimethylsulphoxide (DMSO), we also investigated the impact of DMSO. Although there was no statistical difference considering overall growth between groups of cultivated embryos proper, growth was always maximal in controls and minimal in the medium with TSA (Figure 5A). Notably, the percentage of differentiated neural tissue was significantly lower with TSA than in controls. Other tissues differentiated equally in all groups of cultivated embryos proper (Figure 5B).

Therefore, it may be concluded that both HDACis, VPA, and TSA impair neural growth and differentiation of neural tissue in cultivated embryos proper.

### 2.3. Cell Proliferation and Apoptosis in Embryos Proper Cultivated with VPA

**Figure 6:**
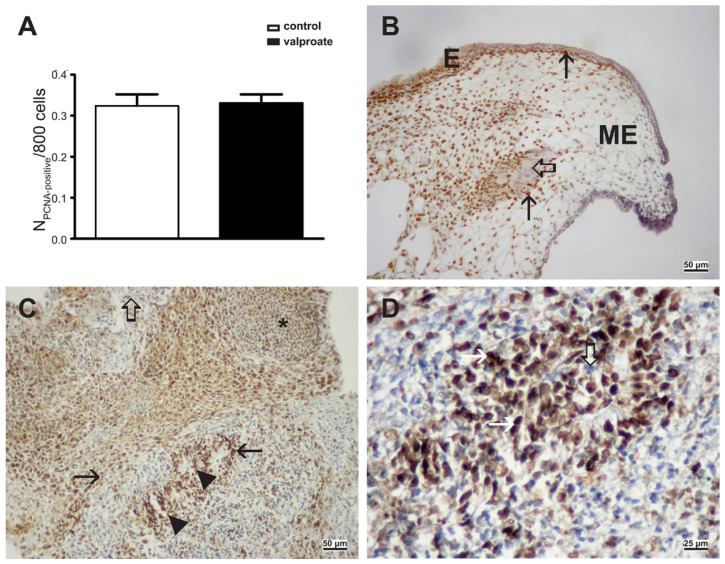
Proliferating cell nuclear antigen (PCNA) expression in embryos cultivated with 1 mM VPA for 14 days. (**A**) PCNA index, arithmetic mean ± SEM. Student *t*-test, *p* > 0.05. (**B**) PCNA-positive cells in controls without VPA (arrow), negative inner control (hollow arrow), epidermis (E), mesenchyme (ME). (**C**) PCNA-positive cells in embryos treated with VPA. Immature neural tissue (arrowhead), pre-cartilage, mesenchyme condensation (asterisk), negative inner control (hollow arrow). (**D**) PCNA-positive cells in embryos treated with VPA. PCNA nuclear signal (arrow), negative inner control (hollow arrow). (**B**–**D**) IHC, DAB, contrasted by hematoxylin. Scale bar on insets (**B**,**C**) 50 µm, and on inset (**D**) 25 µm.

**Figure 7:**
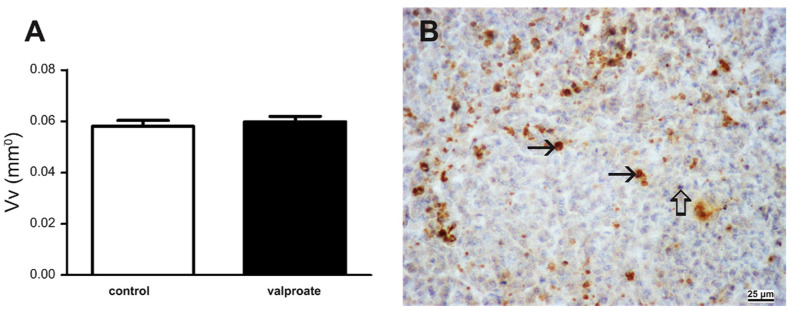
Cleaved caspase-3 expression in embryos cultivated with 1 mM VPA for three days. (**A**) Volume density (Vv), arithmetic mean ± SEM. Student *t*-test, *p* > 0.05. (**B**) Cleaved caspase-3-positive cells (arrows) in embryos cultivated with VPA. IHC, DAB, contrasted by hematoxylin. Scale bar on inset (**B**) 25 µm.

As significantly impaired overall growth was found in embryos grown in vitro with VPA, we were interested in assessing whether VPA could diminish cell proliferation or enhance apoptosis. The cell proliferation marker PCNA and the apoptosis marker cleaved caspase-3 were abundantly expressed in tissues of both groups of embryos at the end of culture (Figure 6 and Figure 7). However, the proliferation index (Figure 6) and the volume density of the apoptosis marker (Figure 7) were similar in control and embryos proper treated with VPA.

Therefore, in our research, cell proliferation was not diminished by VPA, and VPA did not enhance apoptosis to impair overall growth.

### 2.4. Histone Acetylation, DNA Methylation, and Retinoblastoma Protein Expression

**Figure 8:**
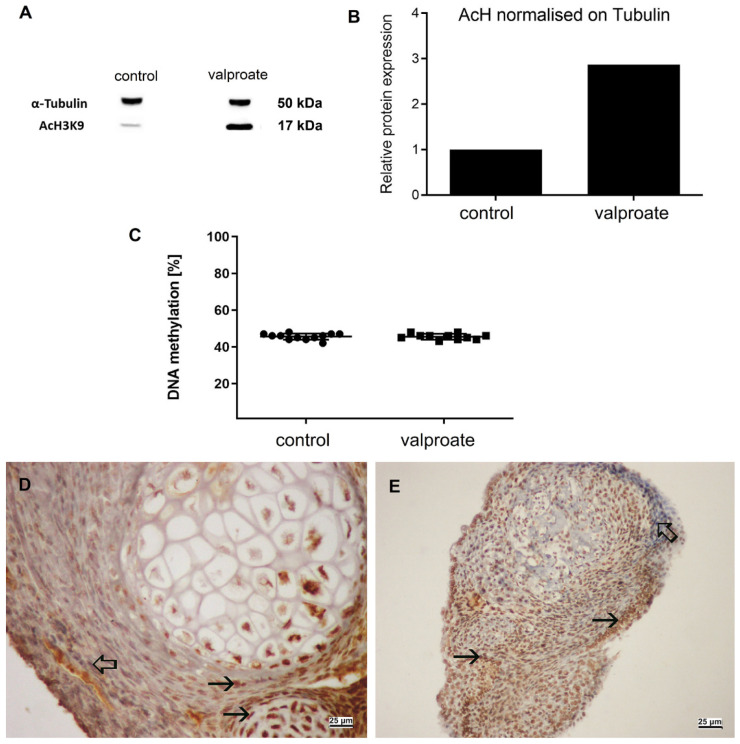
Acetylation of the H3 histone and DNA methylation in embryos proper cultivated with serum for 14 days with or without 1 mM VPA. (**A**) Western blot of α-tubulin and histone H3 acetylated on K9, showing results of control (MEM + serum) and VPA-treated embryos. (**B**) Relative protein expression of acetylated histone 3 on K9 normalized on tubulin. (**C**) Global DNA methylation in embryos proper cultivated for 14 days in vitro with VPA. (**D**) Embryo cultivated without VPA, cells positive on AchH3K9 (arrows), negative inner control (hollow arrow). (**E**) Embryo cultivated with VPA. Cells positive on AchH3K9 (arrows), negative inner control (thick arrow). (**D**,**E**) IHC, DAB, contrasted with hematoxylin. Scale bar on insets (**D**) and E 25 µm.

**Figure 9:**
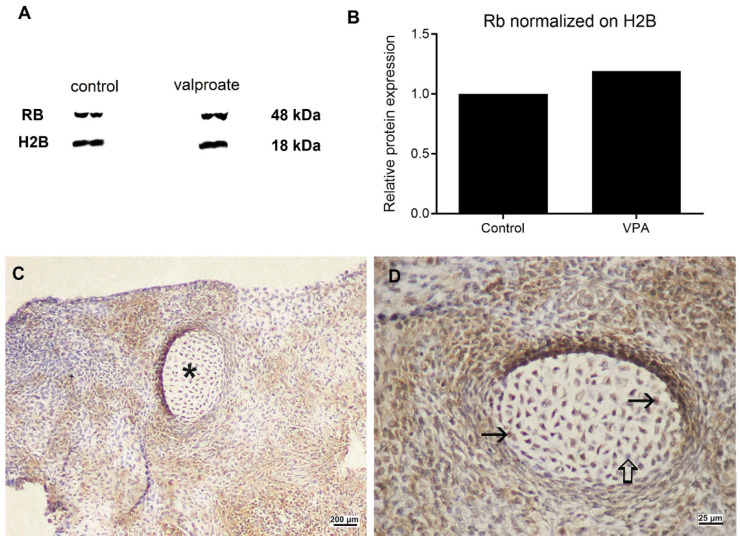
Expression of retinoblastoma protein (pRb) in embryos cultivated for 14 days in vitro with or without 1 mM VPA. (**A**) Western blot. (**B**) pRb expression normalized on H2B. (**C**) pRb-positive signal in embryos cultivated with VPA, cartilage (asterisk). (**D**) pRb (arrow), in embryos cultivated with VPA, negative inner control (thick arrow), detail of (**C**). (**C**,**D**) IHC, DAB, contrasted with hematoxylin. Scale bar on insets (**C**) 200 µm, and on inset (**D**) 25 µm.

As VPA is an HDACi, we wanted to prove its activity by assessing acetylation at lysine9 of the histone H3 (H3AcK9). Indeed, VPA induced acetylation of H3 was 186% higher than in embryos cultivated without VPA (Figure 8A) but did not interfere with another epigenetic mechanism, the global DNA methylation (Figure 8B). As we found a negative impact on embryo growth with VPA but no effect on cell proliferation, we were interested in whether there is a change in the expression of the negative cell cycle regulator, the retinoblastoma protein. Indeed, VPA induced 19% higher expression of the RB protein than in controls (Figure 9A,B). At the single-cell level, RB heterogenous expression was assessed in cells of various tissues (Figure 9C,D).

### 2.5. Transplants of Embryos Proper Pre-Cultivated In Vitro

**Figure 10:**
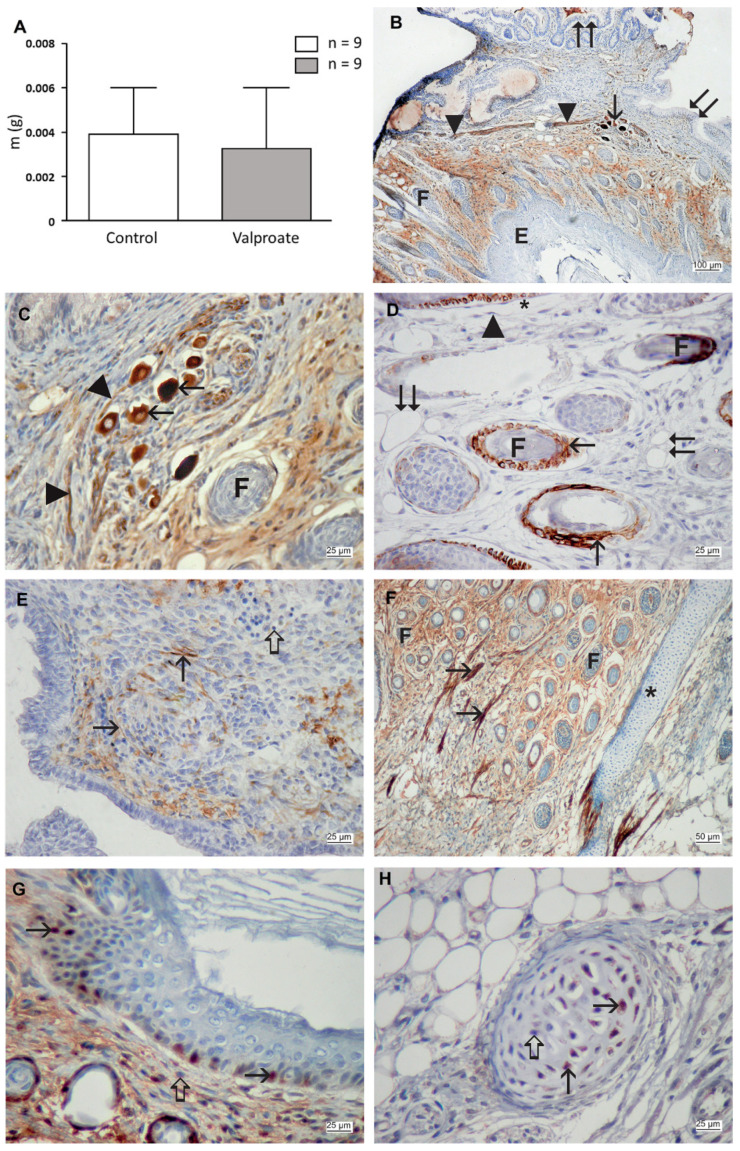
Growth and differentiation of pre-cultivated embryos in transplants under the kidney capsule. (**A**) Embryo weight pre-cultivates without (control) and with 1 mM VPA, arithmetic mean ± SEM, Student *t*-test. (**B**) Control transplant with vegetative ganglion cells positive on NF (arrow), hair follicles (**F**), a bundle of neural filaments (arrowhead); epidermis (**E**), ciliated pseudostratified columnar epithelium (double arrow). (**C**) Control transplant with vegetative ganglion cells positive on NF (arrow), hair follicles (**F**), and neurite (arrowhead). (**D**) Control transplant with cytokeratin 15 expression (arrow) in basal epidermal cells and hair follicles (**F**), adipose cell (double arrow), capillary endothelium (arrowhead), capillary lumen (asterisk). (**E**) Transplant pre-treated by VPA with the expression of nestin (arrow), negative inner control (thick arrow). (**F**) Transplant pre-treated by VPA with the expression of smooth muscle actin (arrow), the hair follicle (**F**), and cartilage (asterisk). (**G**) A transplant pre-treated by VPA. Expression of PCNA in basal cells of the epidermis (arrow). (**H**) Transplant pre-treated by VPA with the expression of pRb in cartilage cells (arrows), negative inner control (thick arrow). (**B**–**H**) IHC, DAB, contrasted by hematoxylin. Scale bar on inset (**A**) 100 µm, on inset (**F**) 50 µm, and on insets (**C**–**E**,**G**,**H**) 25 µm.

To investigate whether the impact of VPA on the growth and neural tissue differentiation is retained in a metabolically richer environment in vivo, we transplanted pre-cultivated embryos proper under the kidney capsule, where they spent additional 14 days. The survival of teratomas from animals treated with VPA (1 mM) was significantly lower than controls (Table 2). The growth of transplants was somewhat lower than that of controls, although the difference was not statistically significant (Figure 10A).

In control transplants, teratoma-like structures continued to differentiate derivatives of the three germ layers (Figure 10B–D). Epidermis developed skin appendages such as hair, while neural tissue differentiated nerves and typical vegetative ganglia with cells with eccentrically positioned nuclei. Neural tissue within transplants was immunohistochemically positive on neurofilaments (NF) and glial fibrillary acidic protein (GFAP). Neural tissue differentiation in transplants pre-cultivated with VPA lagged behind controls, so only nestin as the marker of neural precursors was expressed. At the same time, no positivity on NF or GFAP was found (Figure 10E,F). However, the epidermis developed skin appendages the same way as controls.

### 2.6. Ectoplacental Cones Grown In Vitro/In Vivo

**Figure 11:**
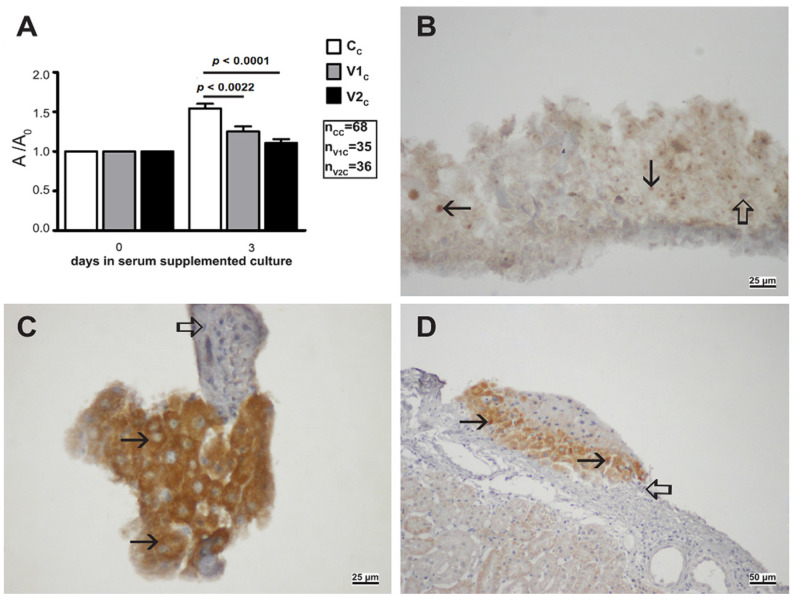
Ectoplacental cone grown in vitro/in vivo with VPA. (**A**) Growth of ectoplacental cones in vitro for three days. Control cones (Cc), cones cultivated with 1 mM VPA (V1c), cones cultivated with 2 mM VPA (V2c), A/A0 Arithmetic mean ± SEM. Mann–Whitney test. (**B**) Expression of CD36 (arrow) cultivated with 1 mM VPA for three days. Negative inner control (hollow arrow). DAB, contrasted with hematoxylin. (**C**) Expression of CD36 in controls pre-cultivated without 1 mM VPA (arrow). Negative inner control (hollow arrow). DAB, contrasted with hematoxylin. (**D**) Expression of CD36 in transplants under the kidney capsule pre-cultivated with VPA (arrow). Negative inner control (hollow arrow). IHC, DAB, contrasted with hematoxylin. Scale bar on inset (**D**) 50 µm, and on insets (**B**,**C**) 25 µm.

As we have shown that HDACi VPA interferes with developmental parameters of the rat embryo proper itself, we additionally investigated the growth of the ectoplacental cone that is the early source of placental development [31] and an extraembryonal part of the whole 9.5 days old gastrulating rat embryo. Our results showed that VPA significantly diminished the growth of the ectoplacental cone grown for three days in vitro, and the dose of 2 mM VPA showed a higher statistically significant difference than 1 mM-dose when compared to controls. In explants, the typical expression of fatty acid translocase CD36 in trophoblast cells [32] was assessed (Figure 11A,B).

Therefore, a higher dose of VPA impairs the development of the ectoplacental cone, similarly to the development of the gastrulating embryo proper.

## 3. Discussion

In line with our previous research [27], the current results showed that VPA significantly impaired neural differentiation of in vitro cultivated gastrulating rat embryos proper (E9.5). These results also show that the negative effect of valproate (VPA) on the development of neural tissue in vitro was retained in subsequent transplants to the metabolically richer environment in vivo in contrast to controls where a higher degree of neural differentiation was assessed histologically and immunohistochemically in the untreated controls. Therefore, a mammalian embryo’s neurodevelopment is already sensitive to VPA at the gastrulation stage, which precedes the stage of the neural tube closure, generally accepted as the target window for neurodevelopmental impairment caused by VPA in humans [1]. Moreover, ectoplacental cones isolated from the same gastrulating 9.5-days-old rat embryos showed significantly lower growth in vitro, showing further evidence of VPA sensitivity at the gastrulation stage. 

The results on the impairment of the embryo’s overall growth in vitro align with our previous results obtained with a higher dose of VPA (2 mM). However, the higher dose also caused a more substantial growth impairment because the embryos treated with 1 mM were approximately twice as big as those treated with 2 mM [27]. These results are in accordance with a rat in vivo study where VPA was applied several days after gastrulation, and the growth of embryos was more impaired with the higher dose [33]. Embryos pre-cultivated with 2 mM in the system used in this research did not develop at all in transplants under the kidney capsule [27]. However, even single germ layers can develop large teratoma-like structures in that site after 14 days, as reviewed before [21]. Notably, the lower dose of VPA (1 mM) did not abolish the remaining potential for teratoma growth in transplants. Therefore, in contrast to previous research [27], a lower VPA dose seems inadequate for the VPA’s antitumor activity that has been of significant interest for the therapy of solid tumors [29].

The 1 mM VPA dose applied to embryos in vitro is similar to the 800 mg daily human dose, and the 2 mM VPA dose is approximately a 1700 mg daily human dose. It was found that, for mothers treated with VPA doses higher than 800 mg daily during pregnancy, their children continued to experience significantly poorer cognitive development than control children were. In comparison, cognitive development seems better in children exposed to VPA doses lower than 800 mg daily [34,35,36]. Although the in vitro model we used deals with a different mammalian species, the negative influence of VPA on early mammalian neural differentiation was found. Moreover, these changes had a prolonged consequence because neural differentiation was also impaired in transplants of VPA pre-cultivated embryos. Such changes might cause the neurodevelopmental problems associated with VPA treatment also in human pregnancy. A negative influence on neural differentiation must be associated with the HDACi activity because our additional results with Trichostatin A (TSA) also impaired neural differentiation in our system. As a potent second-generation HDACi [37], TSA has also been recently investigated in a clinical trial for safety and tolerability in patients with relapsed or refractory hematologic malignancies [38].

Considering the lower overall growth in vitro of embryos treated with the 1 mM VPA, we neither found enhancement of apoptosis nor decline in the cell proliferation as we did previously with a higher dose of VPA [27]. However, on the last day of culture with VPA, we noticed a 19% enhancement of the retinoblastoma protein (pRb) expression compared to controls. pRb protein is primarily a negative cell cycle regulator [39] highly expressed in, e.g., postmitotic ganglia of the mouse neural retina and differentiated layers of the stratified squamous epithelium. In contrast, its expression in the cycling neural retina cells and stem cell basal layer of the stratified squamous epithelium is minimal or absent [40]. Therefore, maturing cells rather than their progenitors possess the highest levels of pRb, which was later confirmed in adult human tissues [41]. In the in vitro cultures and transplants we confirmed typical heterogeneity of pRb expression in cells of developing tissues. Although the regulation of *Rb* gene expression has not been sufficiently investigated [42], *Rb* gene deletion may enhance the proliferation of various undifferentiated cells [43]. Because it was shown that an HDACi activated expression of T*p21* but not the *Rb* gene expression in a tumor cell line [44], it seemed unlikely that histone acetylation we assessed in complex cultures treated with VPA could significantly activate *Rb* gene expression, similarly to recently published for other suppressor genes in a tumor cell line [9]. However, it is possible that HDACi, through enhanced *P21* gene and p21 protein expression, also enhanced pRb dephosphorylation that blocks the cell cycle, as shown previously [44]. Inactivation of the retinoblastoma gene has recently been associated with high levels of global DNA methylation [45], while histone acetylation by an HDACi could induce DNA demethylation [46]. However, we did not assess any global DNA-methylation changes in VPA-treated embryos compared to controls. The question of pRb expression regulation associated with the growth in the model system used here remains to be answered by a future study, best at the single-cell level to avoid confounding elements such as heterogeneity of its expression in various cells even from the same tissue.

The natural 3D developmental system used in this investigation is superior to the two-dimensional cell culture models from the perspective of preservation of tissue interactions necessary for optimal differentiation [47,48]. Moreover, in this model system, derivatives of all three germ layers simultaneously progress to terminal cell differentiation. Recently developed mammalian gastruloid models seem unable to simultaneously develop the brain, somite, neural tube, gut tube, and beating heart-like structures [19]. Considering the previous research with VPA activity in vitro [27], we showed that VPA directly impairs the potential for neural differentiation of the rat ectoderm as a part of the trilaminar gastrulating embryo. Therefore, the ex vivo system enabled us to research the impact of VPA directly on embryonic development without any interference from the maternal organism. 

By cultivating an isolated 9.5-days-old extra-embryonic ectoplacental cone that represents an early embryonic source of placental development [49], we also showed that valproic acid directly impaired its growth. Therefore, the negative impact of valproic acid on placental development may start before midgestation, as previously published [25]. Recent research on placental development influenced by VPA in vivo has shown mainly the change in the expression of the placental transporters [25]. Another epigenetic drug, a DNA hypomethylating agent applied during gastrulation in vivo, caused changes in glycoprotein expression, a severe decline of placental growth, and disturbance of its structure that was improved when the application was during the later stages of development [50,51]. These results highlight the importance of epigenetic mechanisms for the development of mammalian placenta at gastrulation.

The rat we used in our research is the mammalian species historically most widely used in biomedical sciences and pharmacological drug testing. It possesses important advantages over the mouse for research into causes and treatment of neurodevelopmental disorders such as ASD that are included in the valproate spectrum disorder [52]. We may conclude that this research on the gastrulating rat embryo confirmed the dose-dependent sensitivity to VPA for neural differentiation and development of the early placental source, pointing to an earlier time window for the adverse impact of VPA during mammalian pregnancy than suspected. As for the antitumor therapeutic activity of VPA, these results show that it is also dose-dependent and that VPA may be inadequate for antitumor therapy at the lower dose used in the present research.

## 4. Materials and Methods

### 4.1. Ethical Statement

All animal procedures were conducted according to the Directive 2010/63/EU and those of Croatian Law on the protection of experimental animals. They were approved by the Ethical Committee of the School of Medicine, University of Zagreb, Croatia (No. 04-76/2006-9).

### 4.2. Animals

Inbred rats of the Fischer strain were obtained from the registered Animal Facility for laboratory rodents at the School of Medicine, University of Zagreb, Department of Biology, and kept in standard conditions. Three-month-old females and males were caged together overnight, and if sperm was found in the vaginal smear the next morning, it was considered to be the 0.5-day post-coitus (dpc). At 9.5 dpc (E9.5), rat dams were anesthetized with 0.8 mL/kg of ketamine (Narketan^®^; Vétoquinol, Bern, Switzerland) and 0.6 mL/kg of xylazine (Xylapan^®^; Vétoquinol, Bern, Switzerland). Deciduae were isolated from the uteri using a dissecting microscope, and egg cylinders were removed with watchmaker’s forceps. Reichert’s membranes were removed from egg cylinders, and they were cut at the level of the amnion. Thus, the gastrulating embryo propers were isolated, consisting of only three germ layers (ectoderm, mesoderm, and endoderm). The ectoplacental cones were isolated from other extra-embryonic parts that were discarded.

### 4.3. In Vitro Culture

A stainless steel grid was placed and covered by lens paper in a 60 × 15 mm center-well organ culture dish (BD Falcon™, Oxford, UK). Embryos proper or ectoplacental cones were plated on the lens paper wetted by the medium poured into the central well. Embryos proper were grown in Eagle’s Minimum Essential Medium (MEM) with Hank’s balanced salt solution with 50% rat serum (controls) or in the same medium with 1 mM valproate (VPA, valproic acid sodium salt, P4543; Sigma Aldrich St. Louis, MO, USA) or Trichostatine A (T8552 Sigma Aldrich, St. Louis, MO, USA) 66 nM dissolved in DMSO (D8418, Sigma Aldrich, St. Louis, MO, USA), or DMSO (1.2%). Ectoplacental cones were grown in MEM with 50% serum, adding 1 mM or 2 mM VPA. Male rats were anesthetized with 0.8 mL/kg of ketamine (Narketan^®^; Vétoquinol, Bern, Switzerland) and 0.6 mL/kg of xylazine (Xylpan^®^; Vétoquinol, Bern, Switzerland), and their blood was drawn from the aortal bifurcation, serum retrieved and heat-inactivated [27]. Embryos were incubated for 14 days at 37 °C in 5% CO_2_ and 95% humidified air. Media were changed five times starting from the third day of culture.

### 4.4. Survival

Samples that disappeared during cultivation or were completely necrotic upon histological inspection at the end of the culture period were categorized as not surviving [53].

### 4.5. Overall Growth

The overall growth of embryos was noninvasively measured throughout the culture period. By the ocular micrometer, major and minor diameters were measured, and the ellipse area was calculated (A = π × major diameter × minor diameter/4). All values were normalized to the initial measure, which was the measure of overall growth (A/A0) [53]. A/A0 was 1 for day 0 when embryos were first plated.

### 4.6. Transplants In Vivo

Embryos cultivated in vitro for 14 days were transplanted to the ectopic site under the kidney capsule of adult Fischer male rats. Rats were anesthetized with 0.8 mL/kg of ketamine (Narketan^®^; Vétoquinol, Bern, Switzerland) and 0.6 mL/kg of xylazine (Xylpan^®^; Vétoquinol, Bern, Switzerland), and the kidney was visualized through a paravertebral incision. Using a Graeffe’s knife, a small incision was made on the kidney capsule, and a “pocket ”was made beneath. Embryos were transferred to the “pockets” with a braking pipette. The surface wounds were closed with 16 mm Michel’s clamps. Transplants were grown in vivo for another 14 days.

### 4.7. Histology and Immunohistochemistry

In vitro and in vivo grown samples were fixed for 24 h in mild Sainte Marie solution (1% glacial acetic acid in 96% ethanol), dehydrated, and embedded in paraffin. Serial sections (5 μm) were made for routine histology or immunohistochemistry (IHC). For the analysis of survival and differentiation, hematoxylin-eosin stained sections were used as previously described [53]. Survival was calculated as the number of teratomas with recognizable cells present at the end of the 14-day culture period, while the incidence of differentiated tissues was expressed as the percentage of the number of samples. 

Microscopic slides were deparaffinized, cleared in xylene, and hydrated to TBS in graded alcohol solutions for the indirect immunohistochemical method. Antigen retrieval was carried out in Dako Retrieval Buffer pH = 6.0 using a microwave oven on 700 W. After boiling, slides were cooled for 1 min (3×), then cooled for 20 min, blocked with peroxidase blocking reagent (0.03% H_2_O_2_) for 20 min, and rinsed in TBS. Primary antibodies were diluted in 1% BSA/TBS/0.05 Tween20 and applied overnight at 4 °C. Primary antibodies used were mouse anti-human proliferating cell nuclear antigen (PCNA) monoclonal antibody (1:100, Clone PC-10, M0879, RRID: AB_2160651, Dako, Glostrup, Denmark), monoclonal rabbit antibody on cleaved caspase-3 (1:200, 9661, RRID: AB_2341188, Cell Signaling Technology, Inc., Danvers, MA, USA), monoclonal rabbit antibody on neurofilament (NF) (1:8000, ab40796, RRID: AB_2149620Abcam, Cambridge, UK), monoclonal rabbit antibody on hepatocyte nuclear factor 4α (HNF4α) (1:1000, ab41898, RRID: AB_732976, Abcam, Cambridge, UK), monoclonal antibody on cytokeratin 15 (1:1000, ab52816, RRID: AB_869863, Abcam, Cambridge, UK), monoclonal rabbit antibody on nestin (1:1000, ab6142, RRID: AB_305313, Abcam, Cambridge, UK), polyclonal rabbit antibody on retinoblastoma protein (Rb) (1:75, ab6075, RRID: AB 305280, Abcam, Cambridge, UK), monoclonal rabbit antibody on glial fibrilary acidic protein (GFAP) (1:500, ab68428, RRID: AB 1209224, Abcam, Cambridge, UK), monoclonal mouse antibody on CD36 (1:200, AV48129, RRID: AB 1846299, Sigma-Aldrich). The polyclonal rabbit antibody on acetylated histone (H3K9ac) (1:250, ab10812, RRID: AB 297491, Abcam, Cambridge, UK) was applied for only 8 min. Slides were washed 5 × 5 min with TBS and incubated (45 min) with DAKO Dual Link HRP conjugated secondary antibody (K4063, Dako, Glostrup, Denmark). Signal visualization was done with DAB (3,3′-diaminobenzidine) and chromogen-substrate complex for 1 min and stopped in distilled water (dH_2_O). Slides were counterstained with hematoxylin, washed in tap water for 20 min, and covered with glycerol/TBS solution (1:1).

### 4.8. Stereology

Nikon Alphaphot binocular light microscope (Nikon, Vienna, Austria) with Weibel’s M42 test system, made of 42 short test lines, each with two ends as test points at 400 × magnification, was used. Cleaved caspase-3 signals were quantified using the stereological parameter volume density (V_V_). Points of the test system which hit stained nuclei and points which hit the reference space (hits on any part of a section) were counted, and the ratio between the hits falling in stained nuclei (Pi) and hits falling in the reference space (Pt) (V_V_ = Pi/Pt) was calculated and expressed in mm^0^ (mm^3^/mm^3^). The stereological orientation measurement was carried out to define the number of fields to be tested [27]. At least 90 fields per group were assessed.

### 4.9. Proliferation Index

For analyzing the PCNA expression, six samples per group were randomly chosen and serially sectioned. 4 non-adjacent sections were scored in 800 cells at 1000× magnification for each sample. Every DAB-stained nucleus was considered positive, irrespective of the staining intensity. The proliferation index was the number of PCNA-positive cells in 800 cells [54].

### 4.10. Tissue Homogenization

Samples from the VPA-treated and control group were placed in two separate collection tubes with 100 μL of RIPA buffer (Tris-HCl 50 mM, pH 8.0, NaCl 150 mM, SDS 0.1%, Na deoxycholate 0.5%, Triton X-100 1%, 0.5 M EDTA 1% and 4% complete EDTA-free protease inhibitor (COEDTAF-RO, Roche, Basel, Switzerland) and 10 sterile glass beads 1.0–1.51 mm in diameter (Retsch GmbH, Haan, Germany). A bead-based homogenizer (Bartin) was used for homogenization for 2 min at 5000 rpm.

### 4.11. SDS Electrophoresis

Bicinchoninic acid assay (BCA) (Sigma, BCA1) using an Uvikon-860 spectrophotometer (Kontron Instruments), according to the manufacturer’s instructions, was used to assess protein concentration. Tissue lysate was mixed with ¼ Laemmli buffer (Tris HCl pH 6.8, 0.125 M, glycerol 20%, 2-mercaptoethanol 10%, SDS 4%, bromophenol blue 0.004%), boiled (5 min) and centrifuged at 16,000× *g* (1 min). Per well of the gel, 10 μg of protein was loaded, and electrophoresis was run in the Mini-PROTEAN Tetra cell system (Bio-Rad, Hercules, CA, USA) alongside a protein marker (Precision Plus Protein™ Kaleidoscope™ Standards, Bio-Rad, #1610375). 

### 4.12. Western Blot

Western blotting was done in a Mini Trans-Blot Cell (Bio-Rad, 1660828EDU) and PVDF Immobilon membrane (Millipore, Burlington, MA, USA) using buffer formulations and run times accordingly to the general protocol for Western blotting by Bio-Rad. The membrane was blocked (1 h) in 3% BSA in TBST (20 mM pH 7.5 Tris-HCl, 150 mM NaCl and 0.1% Tween 20), rinsed with TBST, and incubated overnight at 4 °C. Primary antibodies to histone H3 acetyl K9 (H3AcK9) (1:500, ab10812, Abcam), α-tubulin (1:10,000, ab52866, Abcam), Rb (1:10,000, ab79416, Abcam), H2B (1:200, sc-8650, Santa Cruz Biotechnology, Dallas, TX, USA) were all in 3% BSA/TBST. Membranes were rinsed with TBST and incubated with the secondary, goat anti-rabbit antibody (1:20,000, ab97051, Abcam, Cambridge, UK) in 3% BSA/TBST) for 1 h at room temperature. Membranes were rinsed and treated with a chemiluminescent dye (Immobilon Western, Millipore) using ChemiDoc XRS+ (Bio-Rad). The signal was quantified using Image Lab™ 6.0 (Bio-Rad). The signal for histone H3 (acetyl K9) was normalized to the housekeeping α-tubulin signal, and Rb-signal was normalized to the housekeeping H2B signal. Obtained values were further normalized to the control values. Results were depicted as a percent difference compared to the control group.

### 4.13. DNA Isolation

At least six samples per group were deparaffinized using xylene (2 × 5 min) followed by rehydration in 100%, 95% and 70% ethanol (3 min each), and, finally, dH_2_O. DNA was extracted in TE buffer pH 9 with 0.1 μg/μL of Proteinase K and 0.25% of Nonidet P40 at 56 °C for 24 h [46]. Next, samples were heated for 10 min at 95 °C to inactivate Proteinase K, spun, and the supernatant was frozen at −20 °C. DNA concentrations and quality were measured with the NanoDrop ND-2000 spectrophotometer (NanoDrop Technologies, Wilmington, DE, USA).

### 4.14. Bisulfite Conversion and Polymerase Chain Reaction

With a clean-up step, one thousand nanograms of genomic DNA were used for bisulfite conversion by EpiTect Plus DNA Bisulfite Kit (#59124; Qiagen, Hilden, Germany). PyroMark PCR Kit (#978703; Qiagen) was used for PCR amplification in the following conditions: 95 °C for 2 min, 43 °C for 90 s, and 72 °C for 60 s for 40 cycles. PCR primers were: forward primer: 5′-GGGTTGGGGATTTAG-3′ and biotinylated reverse primer: 5′-AACCCAAAACCTTA-3′.

### 4.15. Global Methylation Analysis by Pyrosequencing

Global methylation was measured by pyrosequencing. All the steps were performed as recommended by the manufacturer (Qiagen). Pyromark Q24 Advanced System with PyroMark Q24 CpG Advanced Reagents (#970922; Qiagen) was used for the pyrosequencing reaction, as recommended by the manufacturer (Qiagen). The sequencing primer for the rat ID element 5′-GGGGATTTAGTTTAGTGGT-3′ was used [55]. PyroMark Q24 Advanced Software was used to obtain and analyze DNA methylation data.

### 4.16. Statistical Analysis

The normal distribution of the data was tested by the Kolmogorov–Smirnov test, after which parametric or non-parametric tests were chosen to compare variances. The overall growth of embryos and tumor weights were tested by Student *t*-test and the overall growth of ectoplacental cones by Kruskal–Wallis test. For survival and differentiation analysis, proportions of surviving teratomas or differentiated tissues in experimental teratomas were compared by χ^2^ or Fischer’s exact test. Differences in the volume density (V_V_) of anti-cleaved caspase-3 positive cells and proliferation index were analyzed by Student’s *t*-test. The statistical significance level was set at *p* < 0.05.

## Figures and Tables

**Table 1:** Survival in vitro of rat embryos cultivated with valproate (VPA) (1 mM). Fischer’s exact test.

	MEM + Serum (Control)		MEM + Serum + VPA	
	N	%	N	%
Explanted embryos	57	100	30	100
Teratomas developed in vitro	50	87.7	30	100

**Table 2:** Survival in vitro of rat embryos pre-cultivated with VPA. Fischer’s exact test.

	MEM + Serum (Control)		MEM + Serum + VPA	
	N	%	N	%
Transplanted explants	9	100	8	100
Teratomas developed in vivo	9	100	4	50 *

* *p* = 0.0294.
